# Effects of urbanization on resource use and individual specialization in coyotes (*Canis latrans*) in southern California

**DOI:** 10.1371/journal.pone.0228881

**Published:** 2020-02-05

**Authors:** Rachel N. Larson, Justin L. Brown, Tim Karels, Seth P. D. Riley

**Affiliations:** 1 Department of Biology, California State University Northridge, Northridge, California, United States of America; 2 Santa Monica Mountains National Recreation Area, National Park Service, Thousand Oaks, California, United States of America; Universita degli Studi di Sassari, ITALY

## Abstract

Urban environments are unique because fragments of natural or semi-natural habitat are embedded within a potentially permeable matrix of human-dominated areas, creating increased landscape and, potentially, habitat heterogeneity. In addition, urban areas can provide diet subsidies for wildlife species in the form of fruiting ornamental plants, trash, and domestic animals. Ecological opportunity in the forms of habitat and food heterogeneity are thought to be important mechanisms in maintaining individual specialization. Identifying which contexts, traits, and mechanisms determine the success or failure of individuals within an urban wildlife population could potentially provide predictions about which populations may succeed in human-dominated landscapes and which may experience local extinction. We used both scat and stable isotope analysis of whiskers to investigate the degree to which coyotes (*Canis latrans*) utilized anthropogenic subsidies and exhibited individual diet specialization across the urban-rural gradient in southern California. Land use surrounding scat and isotope sample locations was also evaluated to determine the effect of land cover on diet. Human food constituted a significant portion of urban coyote diet (22% of scats, 38% of diet estimated by stable isotope analysis). Domestic cats (*Felis catus*) and ornamental fruit and seeds were also important items in urban coyote diets. Consumption of anthropogenic items decreased with decreasing urbanization. In suburban areas, seasonality influenced the frequency of occurrence of anthropogenic subsidies with increased consumption in the dry season. The amount of altered open space (areas such as golf courses, cemeteries, and landscaped parks) nearby had a negative effect on the consumption of anthropogenic items in both urban and suburban areas. Contrary to our hypothesis, urban coyotes displayed reduced between-individual variation compared to suburban and rural coyotes. It is possible that the core urban areas of cities are so densely developed and subsidized that wildlife inhabiting these areas actually have reduced ecological opportunity. Suburban animals had the broadest isotopic niches and maintained similar individual specialization to rural coyotes. Wildlife in suburban areas still have access to relatively undisturbed natural areas while being able to take advantage of anthropogenic subsidies in neighboring residential areas. Therefore, areas with intermediate urban development may be associated with increased ecological opportunity and specialization.

## Introduction

As humans increasingly develop and inhabit urban areas, the number of species coming into contact with humans is increasing [1,2]. Species response to urbanization varies, with some increasing and some decreasing their activity in urban areas [3,4]. Dynamic urban settings provide opportunities to study how individual-level variation in resource use influences the successful persistence of a population of a synanthropic species (i.e., one that lives near and benefits from humans; [5–7]). Identifying which contexts, traits, and mechanisms determine the success or failure of individuals within an urban wildlife population is important from a conservation perspective and could potentially provide predictions about which populations may succeed in human-dominated landscapes and which may experience local extinction [3,5].

Intra-population variation is a widespread phenomenon with important implications for the ecology, evolution, and management of a species. Heterogeneity in resource availability has been identified as a possible mechanism for promoting and maintaining individuality in diet [8–13]. For example, Robertson et al. (2015) found that European badger (*Meles meles*) groups with a higher proportion of high-quality foraging habitat in their home ranges had lower levels of individual specialization than groups with a lower proportion of high-quality habitat. Access to multiple food webs can also increase specialization between individuals. In coastal British Columbia, wolves (*Canis lupus*) living on near-shore islands have access to terrestrial and marine food webs and exhibit the strongest evidence for individual specialization compared to wolves living on the mainland (access to a terrestrial food web only) or on off-shore islands (access to a marine food web only; [12]). If maintained for a long period of time, individual diet specialization can alter population and community dynamics [6,14,15], and even promote speciation [16].

Urban areas provide a unique ecosystem with ecological opportunity in the form of anthropogenic subsidies to examine the niche variation hypothesis [17], which posits that niche expansions can occur primarily because of increased inter-individual variation in resource use. Ecological release from competition via resource subsidies allows individuals to specialize on one or a few resources that are now more abundant, rather than maintaining a generalist diet consisting of small amounts of scarce resources. In human-dominated environments the other resources available to wildlife may also be altered. In addition to the resources available in natural habitat patches, urban areas may have increased available surface water, may have increased canopy and sub-canopy cover for shelter, and may often provide novel food sources (reviewed in [18]). Anthropogenic food sources directly available to wildlife, such as discarded human food, pet food, human-associated fruits, and domestic animals, are considered primary urban resource subsidies. However, urbanization can also enhance the abundance of native (or non-native) prey species, thus providing a secondary resource subsidy to consumers [19].

Coyotes (*Canis latrans*) represent a model organism to test for individual specialization due to their omnivorous, highly variable diet [20]. This species readily colonizes urban areas and learns to consume the resources found in those areas (reviewed in [21]). Their natural prey base in cities can be limited, as native rodents are sensitive to habitat loss and fragmentation [22,23], however some prey populations can be as abundant in urban areas as in nearby natural areas (e.g., rabbits; [19]). Coyotes may subsidize their diet with anthropogenic items in increasingly urban (i.e., isolated) patches. For example, in the Santa Monica Mountains north of Los Angeles, Fedriani et al. (2001) found that increasing urbanization correlated with elevated consumption of human-related food items. In Chicago nature preserves, Newsome et al. (2015) found evidence for individual specialization, characterized by high between-individual variation and low within-individual variation in diet, suggesting individual coyotes maintained different diets for multiple months. Coyotes are a particularly important species because they often function as an apex predator and are capable of killing pets and even injuring people, particularly children [24,25]—interactions that often elicit strong reactions from the public [21,26,27]. Anthropogenic subsidies have also been linked to conflict between humans and coyotes [28] and therefore are of particular management concern.

The South Coast Ecoregion of southern California is the largest metropolitan area in the United States [29], yet populations of coyotes persist there [30–32]. The region’s Mediterranean climate ensures a year-round supply of anthropogenic subsidies in the form of cultivated edible plants and free-ranging or feral domestic animals. To assess anthropogenic resource use by coyotes in this region, we took the approach of combining traditional scat analysis with concurrent stable isotope analysis. The carbon and nitrogen isotope compositions of animal tissues have been used to quantify diet composition of a myriad of species. Carbon enrichment, the ratio of ^13^C to ^12^C in tissues, is an indicator of the primary producers of a food chain. Studies have shown that processed foods and meats, such as chicken, pork, and beef, consumed by people living in North America have high ^13^C values due to the prevalence of corn and its derivatives (e.g., high-fructose corn syrup; [33,34]). If a coyote consumes this kind of food, these isotopes are assimilated into its tissues, making it possible to detect a proportional representation of anthropogenic resources consumed. Nitrogen enrichment, the ratio of ^15^N to ^14^N, can be used as an indicator of trophic level, or the amount of animal protein consumed, because ^15^N is preferentially retained in consumers and higher trophic levels [35,36]. A previous study has linked low-protein diets to increased human-wildlife conflict in coyotes in Toronto [28]. Where scat analysis identifies short-term and seasonal variations in prey item occurrence, stable isotope analysis represents relative biomass of resources consumed. When paired, both methods provide a more complete picture of diet. We predicted a positive relationship between the consumption of anthropogenic resources and degree of urbanization from rural areas to urban centers. We also predicted that the overall dietary niche of urban coyotes should be larger compared to the dietary niche of suburban and rural coyotes. This increase in niche size should be related to an increasing level of individual specialization in diet in urban coyotes. The increased availability of novel resources, such as anthropogenic food, cultivated edible plants, and domestic animals should drive individuals to specialize on specific resources in urban areas.

## Methods

### Study area

Southern California is characterized by hot, dry summers and cool, wet winters with average daily temperatures of 10.4°C to 19.3°C in December and 16.5°C to 25.1°C in July. Precipitation in Southern California primarily falls in the winter and early spring when daytime temperatures favor the C_3_ photosynthetic pathway [37,38], resulting in a mosaic of coastal sage scrub, chaparral, oak woodland, riparian, and grassland communities dominated by C_3_ plants (δ^13^C ≈ −27‰). Dominant plant species include sages (*Salvia* spp.), California sagebrush (*Artemisia californica*), California brittlebush (*Encelia californica*), toyon (*Heteromeles arbutifolia*), oaks (*Quercus* spp.), manzanitas (*Arctostaphylos* spp.), California lilacs (*Ceanothus* spp.), sumacs (*Rhus* spp.), California buckwheat (*Eriogonum fasciculatum*), and chamise (*Adenostoma fasiculatum*). The coyote population in the Los Angeles area inhabits the second largest and second most populous city in the United States [39]. Coyotes in this system are known to occur throughout all levels of urbanization, from core downtown areas through suburbs to rural areas ([31], JLB and SPDR unpub. dat.). Coyote scats, vibrissae, or a combination of these were collected from coyote populations in Los Angeles (~34°3’0” N, 118°15’0” W; Los Angeles County); the Conejo Valley cities of Thousand Oaks, Westlake Village, and Agoura Hills (~34°11’22” N, 118°52’30” W; Los Angeles and Ventura Counties); and rural Ventura County (~34°25’5” N, 118°55’4”W).

We divided the overall study area into three main areas to coarsely reflect the gradient of urbanization: the “urban study area”, the “suburban study area”, and the “rural study area”. The urban study area was located in the Los Angeles basin, with approximately 70% of the surface area covered by human development (housing units and commercial/industrial areas) and an average human density of >2,500 people/km^2^. Within this highly-developed area there are small fragments of green space, mostly cemeteries and undeveloped hill-tops. The suburban area was located in the Simi Hills and Santa Monica Mountains, with approximately 50% of the surface area covered by human development and an average human density of 850–900 people/km^2^. Naturally-vegetated open space is mainly found on the undeveloped hill-tops and federal, state, and local parks, while residential and commercial areas lie in the valleys. The rural area (from which only coyote vibrissae were collected) was located along SR 126, from Santa Clarita to Oxnard. Approximately 9% of the surface area is covered by human development and the average human density is 470 people/km^2^. The area is in the Santa Clarita River Valley, which is dominated by agriculture within the valley and bordered by large open spaces in the Topatopa and Santa Susana Mountains.

### Scat analysis

Citizen science volunteers collected scats from 30 habitat patches in the urban study area and National Park Service (NPS) interns collected scats from 14 habitat patches in the suburban study area. Scats were not collected from the rural study area due to logistic constraints. Designated transects were established by JLB along prominent dirt roads or trails (hiking trails or wildlife trails) along the length of each habitat patch. Scat surveys were conducted once a month from September 2016 to August 2018 in both study areas, totaling 24 sampling bouts. Similar to other Mediterranean climates, Southern California has two seasons: a cool wet season where average temperatures lower and the majority of precipitation falls, and a hot dry season where average temperatures are warmer and much less precipitation falls. Scats collected from November to April were classified as “wet season” scats, and those collected from May to October were classified as “dry season”.

Transects were continuously sampled from beginning to end and any scat that matched species identification criteria was collected in a paper bag. Citizen science scat collectors and wildlife interns were trained to identify coyote scat using a protocol developed by JLB (dx.doi.org/10.17504/protocols.io.933h8qn [PROTOCOL DOI]). Scats were identified in the field based on morphology (length, width, and shape), odor, color, location, and nearby sign; distinguishing characteristics were developed from scientific publications and field guides with scat descriptions [40–43]. Citizen scientist identifications were verified by NPS wildlife interns before the scats were processed. Any scat that did not match identification criteria, or identified to a species other than coyote, was removed from analysis. Scats were baked at 60°C for 24 hours to kill any parasites, transferred to nylon hose and washed in a commercial washing machine, then dried in a clothes dryer and stored until dissection. Scat processing was carried out by RNL or NPS wildlife interns. Scats were dissected and contents identified by citizen science volunteers trained by RNL and JLB (training protocol available on dx.doi.org/10.17504/protocols.io.933h8qn[PROTOCOL DOI]), as well as RNL and JLB themselves. All scat contents were identified by at least two observers, one of which was either a study author or an NPS wildlife intern. To identify mammals, RNL made samples of hair from each scat into slides to visualize the internal structures of the hair. Slides were viewed under a compound light microscope at 100× magnification and compared to identification keys [44–46]. If present, mammalian molars were also identified by patterns on the occlusal surfaces and root structure [47]. Birds were identified by the presence of feathers, reptiles were identified by the presence of scales, and invertebrates were identified by the presence of chitinous exoskeleton parts. “Anthropogenic items” were defined as food items that were associated with humans in some way and that are not native to southern California, including ornamental fruits and seeds, domestic cats, trash (identified by the presence of food packaging, paper, aluminum foil, glass, or plastic), pet food (identified by the presence of ground-up corn kernel tip caps and hulls, or commercial birdseed [millet, sunflower]), non-native rats (*Rattus* spp.), Eastern fox squirrels (*Sciurus niger*), house mice (*Mus musculus*), livestock and poultry, domestic dogs (*Canis lupus familiaris*), and domestic rabbits (*Oryctolagus cuniculus*).

To determine if land cover affects diet, we calculated the percent of the area surrounding a scat line that belonged to five categories: Commercial/Industrial, Residential, Agriculture, Developed Open Space, and Natural Open Space. The average home range size of resident coyotes in the study area has been reported as 5 km^2^ [31], so we delimited a circular buffer with a radius of 1.26 km around the linear center point of each scat line to approximate a “pseudo-home range” buffer zone of the coyotes sampled at each scat line. Buffer zones were clipped by major freeways (I-5, I-10, U.S. 101, U.S. 405, State Route 23, and State Route 110), which can be movement barriers to coyotes [48]. Any buffer zones that overlapped among sites were assumed to be used by the same individuals and merged into a single polygon. Scat data for these transects were then pooled for analysis. We used the 2005 Land Use classifications [49] to calculate proportions of land cover in each buffer zone (Table 1). Commercial/industrial and residential cover were labeled “Urban”. Developed parks and open spaces (e.g., community parks with landscaped lawns, golf courses, and cemeteries) and agricultural areas were labeled “Altered Open Space”. We used 2010 U.S. Census data [39] to determine the human population density (in increments of 10,000 people/km^2^) in each buffer zone. Population density was used as a proxy for human activity, which coyotes have been documented to avoid [7,31,50,51]. We also calculated the density (km/km^2^) of surface roads in each buffer zone. All spatial analyses were done using ArcMap Desktop 10.6 (Esri Inc., Redlands CA).

**Table 1:** Land use in the scat transect buffer zones. Land use and human development characteristics in 18 urban and 6 suburban scat transect buffer zones. Spatial analysis was done in ArcMap Desktop 10.6 (Esri, Inc.; Redlands, CA) using [49].

Buffer_Zone	Study_Area	Urban^a^	Altered^b^	Roads^c^	Humans^d^
South LA	Urban	0.9751	0.0167	13.3	7.2
Pan Pacific	Urban	0.9600	0.0390	13.3	6.2
Vista Hermosa	Urban	0.9278	0.0142	15.3	10.8
Evergreen	Urban	0.9274	0.0715	13.5	8.6
Northeast LA	Urban	0.8493	0.0055	12.5	3.8
Baldwin Hills	Urban	0.7986	0.0731	7.7	1.7
Silverlake	Urban	0.8103	0.0370	13.3	2.8
Pierce College	Urban	0.8135	0.1677	7.7	1.8
Inglewood	Urban	0.7871	0.2103	11.2	4.0
Elysian East	Urban	0.7614	0.0532	12.2	1.7
Cheviot	Urban	0.7242	0.2726	10.9	2.3
Elephant Hill	Urban	0.6974	0.0037	11.2	3.3
Ascot Hill	Urban	0.6760	0.0284	9.7	2.8
Echo Park	Urban	0.6663	0.0689	10.9	3.7
Los Feliz	Urban	0.6498	0.0279	9.7	4.8
Calvary	Urban	0.6638	0.3279	12.7	5.6
Ballona Wetland	Urban	0.4582	0.0118	7.7	2.7
Upper Franklin	Urban	0.3501	0.0171	5.9	0.3
Pederson	Suburban	0.6117	0.1212	10.4	1.1
Headquarters	Suburban	0.7939	0.0441	8.3	1.5
CLU	Suburban	0.6997	0.0269	8.8	1.6
TO Core	Suburban	0.4524	0.0213	5.2	0.9
Morrison	Suburban	0.3687	0.0105	2.7	0.2
China Flats	Suburban	0.0000	0.0000	1.1	0.1

^a.^ Proportion of the buffer zone’s surface area that was classified as commercial/industrial or residential land use.

^b.^ Proportion of the buffer zone’s surface area that was classified as agriculture or developed parks/other open space land use.

^c.^ Road density in the buffer zone in km per km^2^.

^d.^ Human density in the buffer zone in 10,000 people per km^2^.

We determined both the frequency of occurrence (*FO*) and percentage of occurrence (*PO*) of food items in scats at each site [52–54]. *FO* is calculated as:

$${FO}_{i}(\%)=\frac{n_{i}}{N}\times 100$$

where *n*_*i*_ is the number of scats containing a food item *i* and *N* the total number of scats. Thus, *FO* measures the percentage of scats that contain a given food item. *PO* is calculated as:

$${PO}_{i}(\%)=\frac{n_{i}}{\Sigma n_{i}}\times 100$$

Thus, *PO* measures the contribution of each food item *i* expressed as a percentage of the total number of occurrences of all food items. *PO* will total to 100%. *FO* will total over 100% because numerous scats (N = 919, 59.1% in the urban area; N = 913, 56.8% in the suburban area) contained more than one food item, so that *∑n*_*i*_ >> *N*. Although *FO* and *PO* do not necessarily approximate the volumetric importance of items in the diet, they can provide valuable insight into carnivore ecology [54]. In general, *FO* and *PO* are highly concordant in their rankings of food items in carnivore diets [52–54]. We focus our results summary on *FO* given it is the most frequently used method in diet studies and is readily interpretable [52,54], but also present *PO* to enable comparison with other diet studies that have used this metric.

Additionally, a niche breadth statistic (*B*) was calculated for each site [55]:

$$B=\frac{1}{\Sigma p_{i}^{2}}$$

where *p*_*i*_ is the proportion of food item *i* in the diet as calculated by:

$$p_{i}=\frac{n_{i}}{{\Sigma n}_{i}}$$

To calculate a niche breadth statistic *B*, we evaluated 9 categories of food items that occurred in >10% of scats (FO) across both study areas: birds, reptiles, invertebrates, trash, ornamental fruit and seeds, domestic cats (*Felis catus*), and three native mammalian prey: rabbits (*Sylvilagus* spp.), ground squirrels (*Otospermophilus beechyi*), and pocket gophers (*Thomomys bottae*). *B* can range from 1 (the predator only consumes one type of food item) to 9 (the predator consumes all 9 types of food items in equal frequencies) since there are 9 food item categories.

We used Morisita’s index of niche overlap [56] to determine the degree to which the diets of coyotes from different levels of urbanization and seasons overlap. Morisita’s index, *C*, is calculated as:

$$C=\frac{2\Sigma p_{ij}p_{jk}}{\Sigma p_{ij}(\frac{n_{ij}-1}{N_{j}-1})+\Sigma p_{ik}(\frac{n_{ik}-1}{N_{k}-1})}$$

Where *p*_*ij*_ is equal to the proportion of food item *i* in the diet of population *j*, *n*_*ij*_ is the number of scats in population *j* containing food item *i*, *N*_*j*_ is the total number of scats collected for population *j*, *p*_*ik*_ is the proportion of food item *i* in the diet of population *k*, *n*_*ik*_ is the number of scats in population *k* containing food item *i*, and *N*_*k*_ is the total number of scats collected for population *k*. Niche overlap is a standardized metric, ranging from 0 (no overlap in diets) to 1 (complete overlap in diets).

Finally, to test for the effects of human population density, road density, and amount of human-dominated land surrounding a scat line on the consumption of anthropogenic items, we used analysis of covariance (ANCOVA) with population density, road density, and percent urban and altered open space as covariates and study area (urban or suburban) and season (wet or dry) as factors with two levels. Altered open space and human density were square-root-transformed and urban land and road density were squared to meet assumptions of homogeneity of variance.

### Stable isotope analysis

Coyote vibrissae samples were collected from animals captured alive and recovered dead as part of several ongoing wildlife monitoring projects. Because vibrissae are metabolically inert, their isotopic composition reflects an average of diet consumed during those months. Samples from each individual were stored dry in labeled coin envelopes until analysis. Samples of common (> 10% frequency of occurrence) food categories found in coyote scat from urban and suburban environments were also collected to create a reference dataset for potential food items. Samples of mammalian hair were collected only from scats or stomach contents in which the remains of just one food category was present. Additional mammal samples were collected from museum specimens or opportunistic roadkill. We also collected chitinous exoskeletons of common insect prey items and fruit samples from ornamental plants.

Vibrissae were rinsed in a 2:1 chloroform:methanol solution to remove surface contaminants. Vibrissae were then subsampled into ~0.2–0.3mg segments which were processed individually. Prey hair was cut into small segments (1-2mm) using a razor blade. Insect exoskeletons were first lipid-extracted using three 24-hour washes of chloroform:methanol solution, desiccated for 24 hours, then homogenized with a small mortar and pestle. We used approximately 0.5–0.6mg of sample per each mammal or insect specimen. Plant samples were desiccated in a lyophilizer then homogenized with a small mortar and pestle. We used approximately 1mg of sample per plant specimen. Samples were weighed into tin capsules for δ^13^C and δ^15^N analysis on a Costech 4010 elemental analyzer (Valencia, CA) coupled to a Thermo Scientific Delta V mass spectrometer at the University of New Mexico’s Center for Stable Isotopes for analysis. Isotopic results are expressed as δ values, δ^13^C or δ^15^N:

$$\delta^{13}C\;or\;\delta^{15}N=\left(\frac{R_{sample}-R_{standard}}{R_{standard}}\right)\times 1,000$$

where R_sample_ and R_standard_ are the ^13^C/^12^C or ^15^N/^14^N ratios of the sample and the standard (Vienna Peebee Belemnite [C] or atmospheric nitrogen [N]); the units are expressed as parts per thousand, or per mil (‰).

We used a Stable Isotope Mixing Model in R (package ‘simmr’) to quantify coyote diet composition [57]. This Bayesian model uses the isotope signatures of prey to reconstruct consumer diet. Some of the food items had isotope values that were not statistically different (see below). Rabbits and pocket gophers were combined into the single category “rabbit-gopher”. Therefore the 9 food items used in the niche breadth calculation were reduced to six for the isotope mixing model, which included three sources of natural prey (rabbit-gopher, ground squirrels, and Jerusalem crickets [*Stenopelmatus* sp.]), two sources of anthropogenic resources (human food and domestic cats), and one plant source (figs; *Ficus* spp.).

This model requires tissue-specific isotopic trophic discrimination factors. Typically, trophic discrimination factors represent the isotopic difference between a consumer’s tissue (e.g., vibrissae) and that of its diet, which for carbon isotopes is commonly denoted by Δ^13^C_tissue-diet_. We used a δ^13^C trophic discrimination factor of 1.5‰ for the mixing model [58,59]. We used a δ^15^N trophic discrimination factor of 3.5‰ [58,60–62]. For both δ^13^C and δ^15^N trophic discrimination factors, we used an error estimate (SD) of 0.5‰ in the mixing models. The isotopic composition of human food was estimated from human hair of 14 Southern California residents by using discrimination factors of 2.0 for δ^13^C and 3.5‰ for δ^15^N. We then applied a δ^13^C discrimination factor (Δ^13^C_tissue-diet_) of 2.5‰ for this food source in the mixing model, which is typical of mammalian carnivore keratins [58,59,63].

We used multiple analysis of variance (MANOVA) to assess differences in δ^13^C and δ^15^N in among food sources, followed by a one-way ANOVA with a post hoc Tukey-Kramer HSD; significance was assigned at *p* < 0.01 to reduce Type I error. To test for the effects of land cover on stable isotope ratios, a buffer was also applied to the recorded locations of coyote captures and mortalities, similar to the scat analysis described above. However, each capture or mortality was treated as an independent event and no stable isotope data were pooled even if buffers overlapped. To test for the effects of human density and amount of urban and altered open space surrounding a collection point on the coyote’s isotope ratios, we used a three-way ANCOVA with human density, percent urban, and percent altered open space as covariates and sex (male or female) and study area (urban, suburban, or rural) as factors. Altered open space and human density were log-transformed, and urban land was arcsine-square-root-transformed to meet assumptions of homogeneity of variance.

As a measure of individual within-group variation, we calculated an index of individual specialization; WIC/TNW [64], for δ^13^C and δ^15^N, where WIC is the within individual component and TNW is total niche width. This was calculated for each study area using a one-way ANOVA with individual as a factor, where WIC is the within-group sum of squares and TNW the total sum of squares [65]. This index varies from 0 (higher inter-individual variation, with each individual utilizing a narrow proportion of the group niche) to 1 (no individual variation, with all individuals utilizing the whole group niche). Low values of WIC/TNW, therefore, indicate higher specialization. All statistical analysis were run in R version 3.5.1 [66].

### Ethics statement

Samples were collected under California Department of Fish and Wildlife permit SC-005636. Land accessed for this research was a combination of protected federal and state park land and private property. Access to private property was granted with written consent from the land owner. Coyotes are not a protected species in California.

## Results

### Scat analysis

Of 3,147 coyote scats analyzed, 1,541 were from urban sites (range:5–309 per scat line, mean = 86.1 ± 86.6 SD) and 1,606 were from suburban sites (range:40–1081 per scat line, mean = 267.5 ± 400.7). The dietary niche breadth was 1.4× larger in the urban (*B* = 7.31) compared to the suburban study area (*B* = 5.21). Overlap in diet composition between the two levels of urbanization was high (*C* = 0.735).

Niche breadths also varied seasonally. In the suburban area, niche breadth was 32% smaller in the wet season (4.14) than the dry season (5.98) and the seasons overlapped by a smaller proportion (overlap index *C* = 0.923) than the urban area. In the urban area, wet season (*B* = 7.23) and dry season (*B* = 7.31) niche breadths were similar, and the seasonal diets overlapped by *C* = 0.994. The seasonal trends observed in the suburban area were driven by the increased consumption of ground squirrels (12.0% to 23.7%), reptiles (3.5% to 12.6%) and ornamental fruits and seeds (11.7% to 37.2%) in the dry season.

#### Urban area

**Fig 1:**
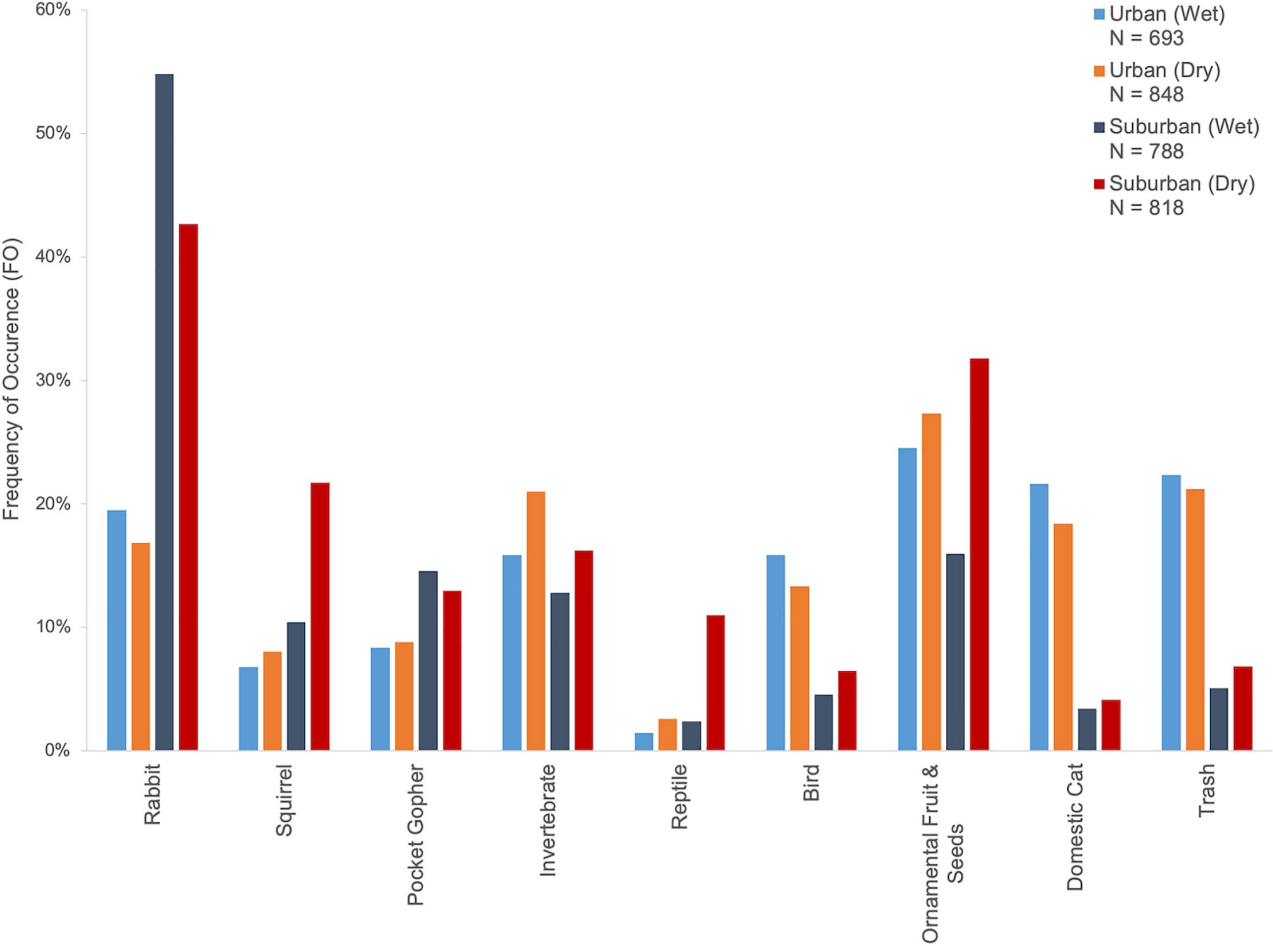
Frequency of occurrence of major diet items in coyote scats. “Urban” and “Suburban” refer to the study area in which the scats were collected and “Wet” and “Dry” refer to the season of collection. *N* refers to the total number of scats dissected during the two year sampling period. “Rabbit” includes cottontail rabbits. “Squirrel” includes California ground squirrels, western gray squirrels (*Sciurus griseus*), and unidentified squirrels (but does not include eastern fox squirrels). “Ornamental Fruit & Seeds” includes plant species not native to California (e.g., figs, palms, and grapes). “Trash” includes items associated with humans (e.g., tin foil, plastic, paper, etc.). Refer to S1 Table for a complete list of all food items.

Anthropogenic items were the most common food item category in coyote scats from sites in the urban area (65.2% of scats), followed by native mammals, invertebrates, birds, reptiles, and native fruits and seeds (Fig 1, S1 Table). Ornamental fruit and seeds (e.g., figs, palm fruits [Arecaceae spp.], and grapes [*Vitis* spp.]) were the most common anthropogenic item, occurring in 26.1% of scats, followed by human trash (22.1%) and domestic cats (19.8%). *Rattus* spp (4.1%) and pet food were uncommon (3.0%). Domestic dogs, eastern fox squirrels, house mice, domestic rabbit, and chickens (*Gallus gallus*) were rarely found in urban scats (≤1.5% of scats, each).

Rodents were the most common native mammals found in urban coyote scats, followed by rabbits. Pocket gophers were the most common rodent, occurring in 8.6% of scats, followed by ground squirrels (7.5%). Native mice (*Peromyscus* spp. and *Reithrodontomys megalotis*, 2.5%) and woodrats were uncommon (1.5%), and only one pocket mouse (*Chaetodipus californicus*) was found. Skunks (*Mephitis mephitis* and *Spilogale gracilis*, 2.7%) and raccoons (*Procyon lotor*, 1.0%) were uncommon in urban coyote scats. Opossum remains were very rare (0.5%). Deer (*Odocoileus hemionus*), shrews (*Soricidae* spp.), and voles (*Microtus californica*) were not found in urban scats.

Invertebrates (18.7% of scats) and birds (14.5%) were also common prey items observed in urban scats. Reptiles (2.1%) and native plants (fruit and seeds; 1.4%) were rarely observed.

#### Suburban area

Native mammals were the most common food item category in coyote scats from sites in the suburban area followed by anthropogenic items, invertebrates, reptiles, birds, and native fruits and seeds (Fig 1, S1 Table). Rabbits were the most common mammals (48.5% of scats), followed by rodents. Ground squirrels were the most common rodent (16.2%), followed by pocket gophers (13.7%), woodrats (7.8%) and native mice species (5.5%). Pocket mice (1.5%) and voles (1.7%) were rarely found. Deer (0.9%), skunks (0.5%), and raccoons (0.4%) were rarely detected in suburban scats. Opossums were found in 0.3% scats and shrews were found in 0.1% scats.

Anthropogenic items were found in 37.2% of scats from suburban sites. Ornamental fruit and seeds were the most common anthropogenic item, occurring in 24.1% of suburban scats. Trash was the second most common anthropogenic item (6.0%). Domestic cats (3.9%), rats (2.4%), and eastern fox squirrels (2.4%) were uncommonly found in urban scats. Pet food (1.8%), house mice (0.6%), chickens (0.4%), and domestic dogs (0.3%) were rare.

Invertebrates were also common prey items, occurring in 14.6% of suburban scats. Reptiles were found in 6.8% of scats, birds were found in 5.5%, and native fruit and seeds were found in 1.4%.

#### Association with land cover

Road density, human density, and percent of urban land were highly correlated (Pearson’s *r*_roads-humans_ = 0.88, *r*_roads-urban_ = 0.81, *r*_humans-urban_ = 0.78; *p* < 0.001 for all correlations) therefore, we used AIC to evaluate non-nested models explaining frequency of anthropogenic foods in diet (Table 2). Assumptions of normality and homogeneity of variance in the response variable were tested and met.

**Table 2:** AIC model selection results for FO of anthropogenic items in coyote scat.

Model	K	ΔAIC	ωAIC	Likelihood
Anthro ~ Study_Area * Season + Altered	3	0	0.748	1.00
Anthro ~ Study_Area * Season + Roads	3	3.88	0.108	0.14
Anthro ~ Study_Area * Season + Humans	3	4.61	0.075	0.10
Anthro ~ Study_Area * Season * Altered	3	5.99	0.037	0.05
Anthro ~ Study_Area * Season + Urban	3	6.29	0.032	0.04
Anthro ~ Study_Area * Season * Altered * Roads	4	26.57	< 0.001	< 0.001
Anthro ~ Study_Area * Season * Altered * Urban	4	30.41	< 0.001	< 0.001
Anthro ~ Study_Area * Season * Altered * Humans	4	30.95	< 0.001	< 0.001

AIC results for model selection used to identify the best model(s) to predict the effect of land use and seasonality on anthropogenic food consumption in coyote scats. “Anthro” = frequency of occurrence (FO; see above for a more detailed definition) of anthropogenic items in coyote scats; “Study_Area” = urban or suburban study area; “Season” = wet (Nov-Apr) or dry (May-Oct) season; “Altered” = percent of buffer zone that is altered open space (e.g., golf courses, cemeteries, city parks); “Roads” = km/km^2^ of roads in the buffer zone; “Humans” = human density (10,000 people/km^2^) in the buffer zone; “Urban” = percent of the buffer zone that is urban land (commercial/industrial and residential land uses).

**Fig 2:**
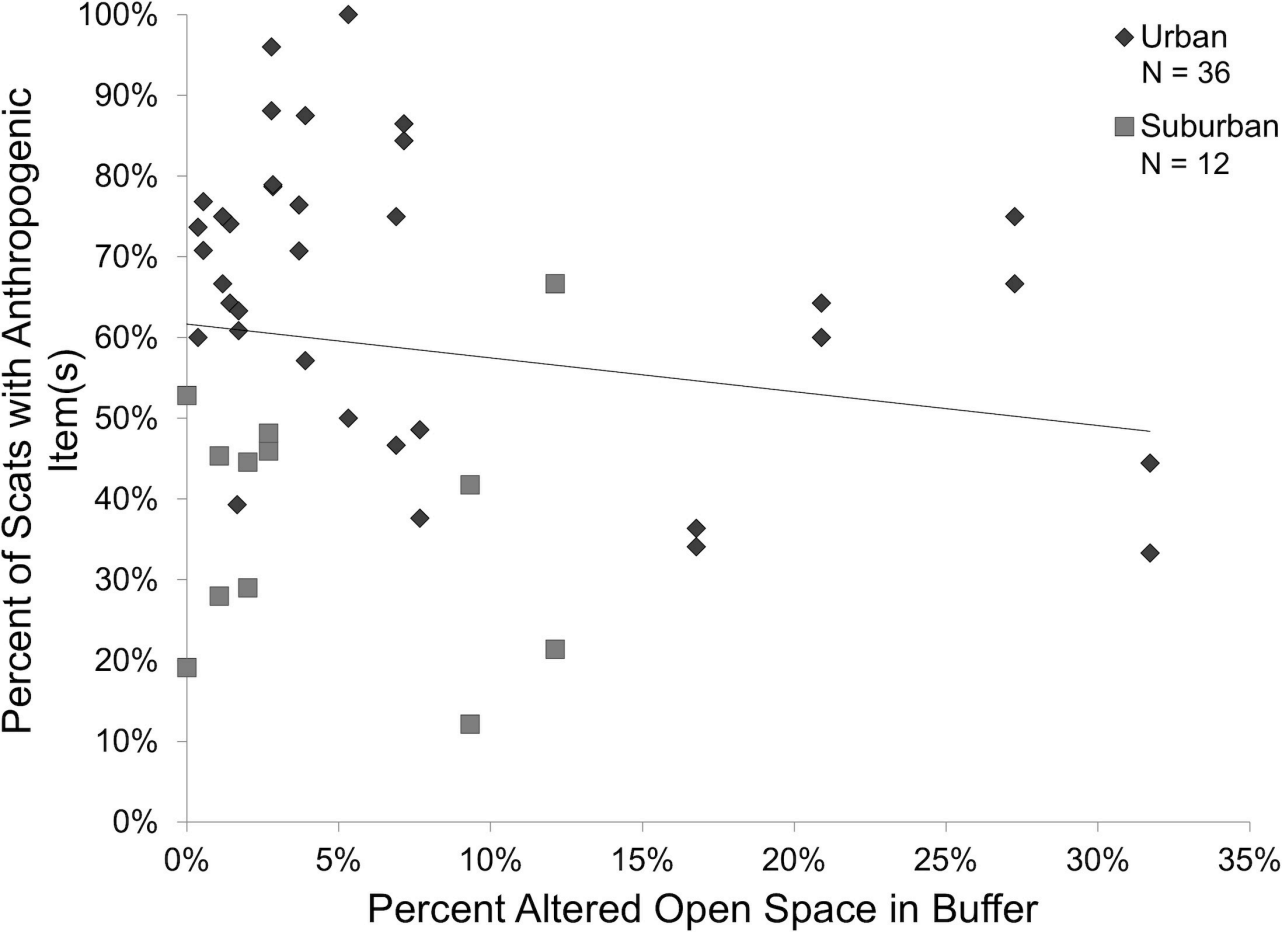
Land use effects on the occurrence of anthropogenic items in scat. The percent of the buffer zone around a scat line that is altered open space cover and the percent of scats collected from that line that had anthropogenic item(s). *N* refers to the total sample size for the study area (each scat transect has two data points: one for wet season frequency of occurrence and one for dry season frequency of occurrence). *Solid black line* represents a linear best-fit model.

The best model (ωAIC = 0.748) explaining frequency of occurrence of anthropogenic items was the model incorporating an interaction between study area and season (*F*_1,43_ = 8.70, *p* = 0.005) with an additive effect of the percentage of altered open space in the coyote’s buffer zone. In the urban area, the average percent of scats containing anthropogenic items was 69.0% (± 4.4% SD) in the wet season, while in the dry season it was 62.9% (± 3.8%). This contrasts with the suburban area, where the average percent of scats containing anthropogenic foods in the wet season was 25.9% (± 4.7%) and in the dry season was 49.9% (± 3.7%). Overall, sites that had more altered open space in their buffer had lower occurrence of anthropogenic items in scats (*F*_1,43_ = 5.87, *p* = 0.02; Fig 2).

### Stable isotope analysis

We collected vibrissae samples from 58 coyotes, 29 males and 29 females. Three coyotes were sampled twice—once when they were captured alive and fitted with a radio collar and again when the collar was retrieved after mortality. The shortest interval between these events was three months. A total of 61 vibrissae were analyzed: 29 vibrissae from urban coyotes, 21 vibrissae from suburban coyotes, and 7 vibrissae from rural coyotes. The average number of subsamples per vibrissa (i.e., replicates per individual) was 16 ± 7 SD (S2 Table).

Isotope signatures of particular prey species (e.g., ground squirrels or rabbits) were not significantly different between study areas (*F*_5,65_ = 0.6, *p* = 0.8). Therefore, we used samples collected from all areas to calculate mean isotope values and associated variance (SD) of potential foods. We found significant differences in δ^13^C and δ^15^N values among potential food sources (δ^13^C: *F*_5,63_ = 58.5, *p* < 0.001; δ^15^N: *F*_5,63_ = 8.2, *p* < 0.001). For δ^13^C, domestic cats (–16.7 ± 1.7‰, *n* = 12) and humans (–18.9 ± 0.9‰, *n* = 14) had higher mean (± SD) values than all other food sources. Ground squirrels (–22.7 ± 0.9‰, *n* = 12), rabbits (–23.0 ± 2.0‰, *n* = 12), and pocket gophers (–23.6 ± 2.2‰, *n* = 11) had similar δ^13^C values. Jerusalem crickets (–25.9 ± 1.1‰, *n* = 10) had values lower than mammals, but higher than plant material. Figs (–28.3 ± 2.2‰, *n* = 7) had the lowest δ^13^C value. For δ^15^N, humans (8.5 ± 0.7‰) had higher mean values than all other food sources. Ground squirrels (6.6 ± 1.4‰) and domestic cats (6.6 ± 0.74‰) had similar δ^15^N values that were higher than other food sources. Figs (5.5 ± 3.4‰), pocket gophers (5.0 ± 1.3‰), and rabbits (4.9 ± 2.1‰) had similar δ^15^N values. Jerusalem crickets had the lowest δ^15^N value (3.6 ± 1.6‰).

**Fig 3:**
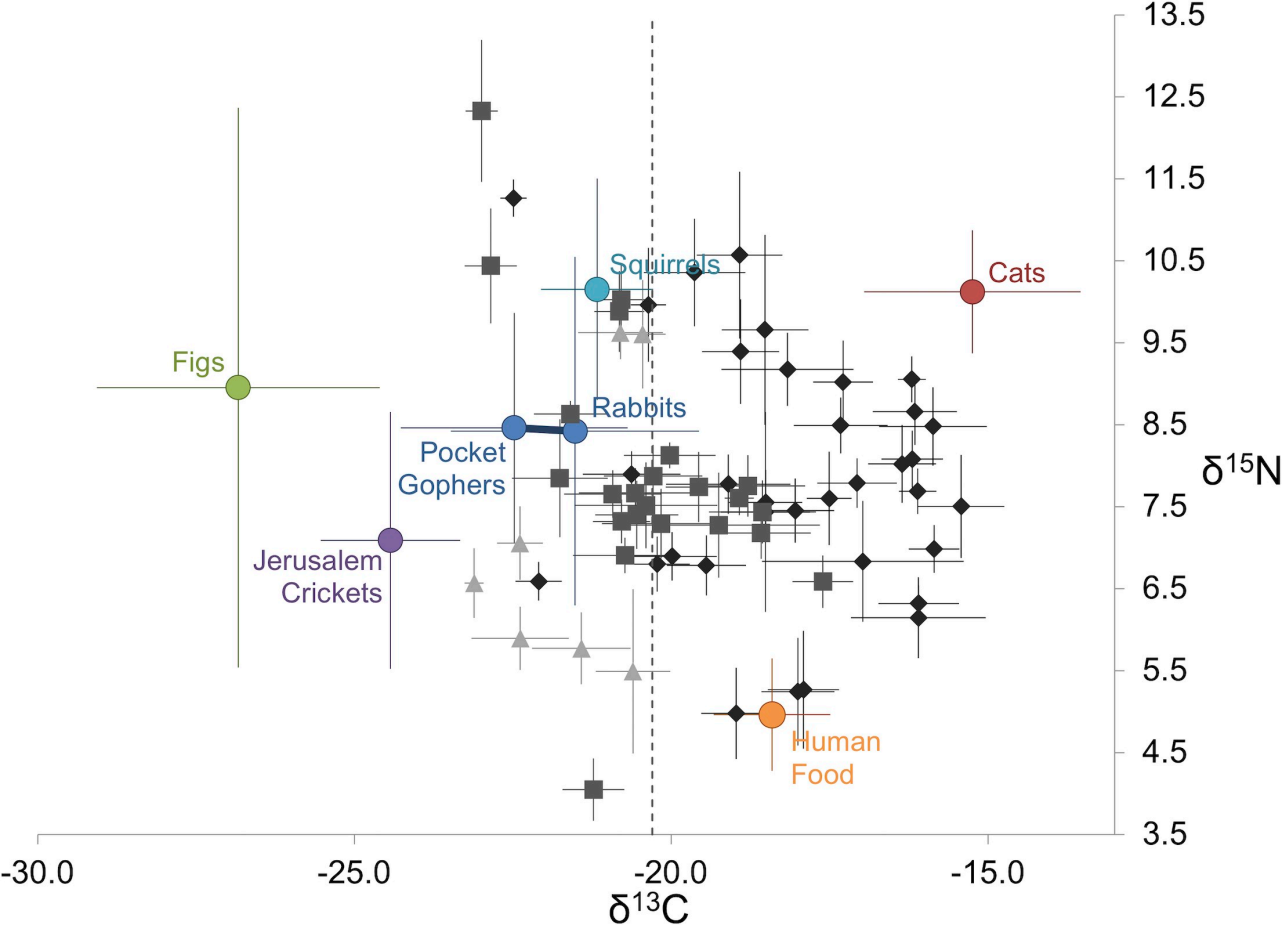
Stable isotope signatures of coyotes and their diet items. Vibrissa δ^13^C and δ^15^N for coyotes (*gray symbols*) and potential food (*colored circles*); error bars represent standard deviation. Sample sizes for coyote populations are shown in parenthesis. Isotope values of potential food items have been corrected for trophic discrimination by adding 1.5 ‰ from measured δ^13^C and 3.5‰ from measured δ^15^N. *Dashed vertical line* denotes approximate δ^13^C threshold of natural versus anthropogenic resources. Mean isotope values for human food were estimated by subtracting 2.0 ‰ from δ^13^C and 3.5‰ from δ^15^N values measured from human hair. Rabbits and pocket gophers appear in the same color because their mean isotope values have been combined for statistical analyses.

Individual coyotes occupied a large portion of the δ^13^C versus δ^15^N isotopic prey space defined by the isotope values of potential foods (Fig 3). We chose a threshold δ^13^C of –20.3‰ to differentiate natural from anthropogenic resources defined by the standard deviation of the natural prey source with the highest mean δ^13^C value (ground squirrels; [67]). This is theoretically the maximum δ^13^C value a natural prey item can hold. This threshold is not meant to represent a stark transition between food sources, but rather a transition from consuming more natural to more anthropogenic food sources. We estimated the isotope values of human food by subtracting 2.0 and 3.5‰ from measured human hair isotope values, which yielded mean (±SD) δ^13^C and δ^15^N values of −20.9 ± 0.9‰ and 4.9 ± 0.7‰ for this potential food source [67]. Therefore, human foods have a unique isotope value relative to other sources of prey available to coyotes in Los Angeles. This value is also similar to other isotopic measurements of North American fast food [33,34] and similar to the “human food” reported for similar study in Chicago (δ^13^C = -20 ± 0.9‰, δ^15^N = 5.5 ± 0.6‰; [67]).

**Fig 4:**
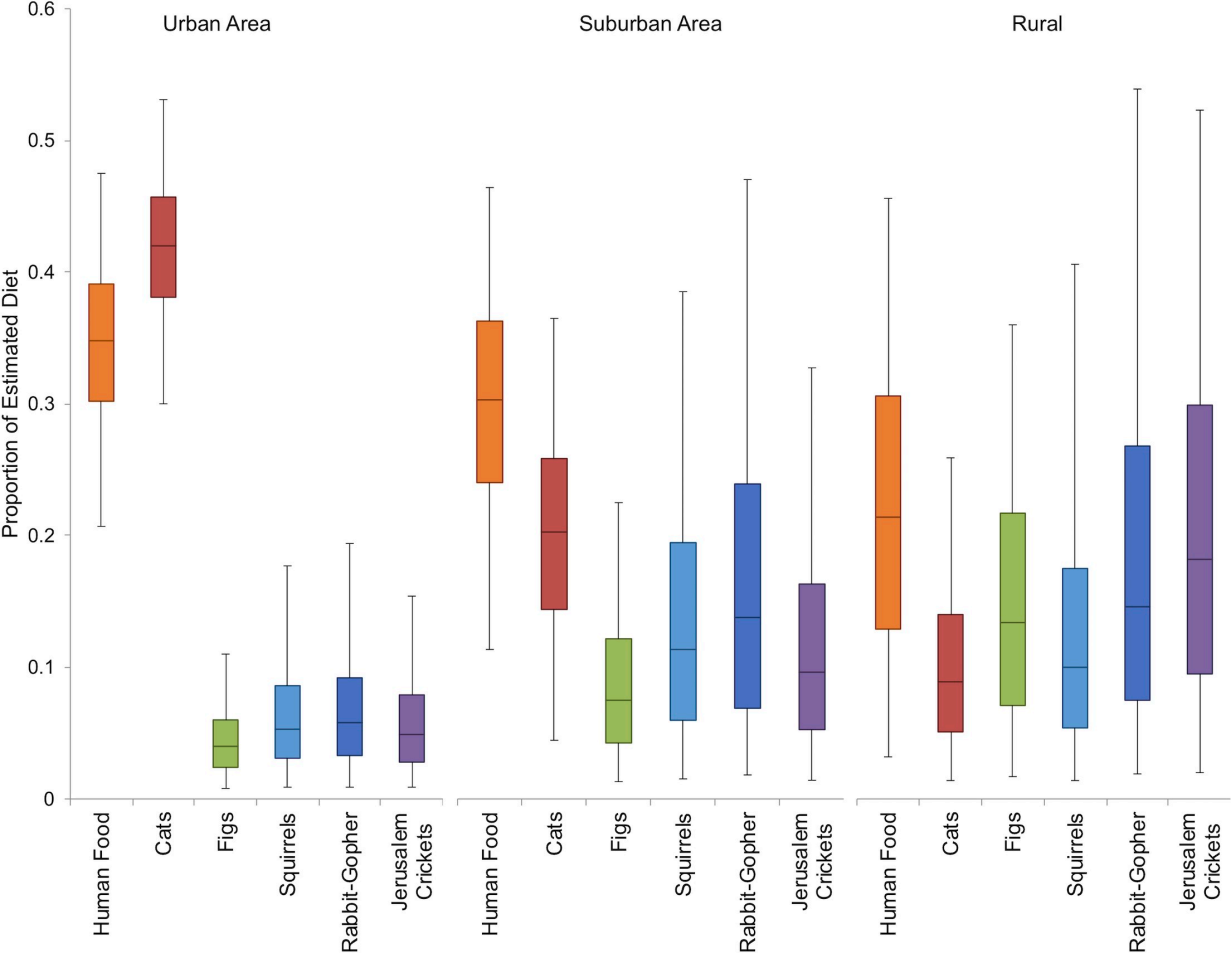
Coyote food item diet proportions as determined by stable isotope mixing models. Boxes represent the second and third quartiles and whiskers denote the first and fourth quartiles as estimated by the SIMMR model. “Rabbit-gopher” refers to the combined rabbit and gopher isotope source.

Most individuals (86%) from the rural area had δ^13^C values below the -20.3‰ threshold, indicating they primarily consumed natural prey. In contrast, approximately 43% of suburban individuals and approximately 90% of urban individuals had mean δ^13^C values that were higher than the threshold. The mean (± SD) proportion of human food in the diet of urban coyotes was 37.6% ± 6.3%, followed by suburban coyotes at 33.8% ± 8.2%, and rural coyotes at 27.3% ± 12.8% (Fig 4). The proportion of domestic cats in the diet decreased as urbanization decreased: 39.5% ± 5.8% of the urban diet, 15.8% ± 7.1% of the suburban diet, and 9.3% ± 6.5% of the rural diet. Figs, the most commonly consumed ornamental plant, increased with decreasing urbanization: figs constituted 5.6% ± 3.1% of urban diets, 11.7% ± 6.9% of suburban diets, and 25.6% ± 10.4% of rural diets. Native prey items also increased as urbanization decreased. In urban diets, rabbit-gophers constituted 9.0 ± 5.8%, followed by squirrels (8.3% ± 5.2%). In suburban diets, rabbit-gophers accounted for 22.7% ± 14.7%, followed by squirrels (16.0% ± 10.9%). In rural diets, rabbit-gophers accounted for 23.1% ± 16.9%, followed by squirrels (14.7% ± 12.0%).

### Association with land cover

For individual coyotes, we found that human density and percent urban land were correlated in their buffer zones (Pearsons *r*_humans-urban_ = 0.58, *p* < 0.05). We used AIC to evaluate non-nested models explaining carbon and nitrogen enrichment in individual coyotes (Table 3, Table 4). Assumptions of normality and homogeneity of variance in the response variables were tested and met.

**Table 3:** AIC results for predicting carbon enrichment.

Model	K	ΔAIC	ωAIC	Likelihood
dC ~ Study_Area	1	0	0.817	1.000
dC ~ Study_Area * Sex + Altered	3	4.82	0.073	0.090
dC ~ Study_Area * Sex	2	5.05	0.065	0.080
dC ~ Study_Area * Sex + Urban	3	6.99	0.025	0.030
dC ~ Study_Area * Sex + Humans	3	7.47	0.020	0.024
dC ~ Study_Area * Sex * Altered	3	15.91	< 0.001	< 0.001
dC ~ Study_Area * Sex * Urban	3	17.20	< 0.001	< 0.001
dC ~ Study_Area * Sex * Humans	3	17.99	< 0.001	< 0.001
dC ~ Altered	1	30.39	<0.001	< 0.001
dC ~ Sex	1	35.31	<0.001	<0.001
dC ~ Study_Area * Sex * Altered * Humans	4	45.16	<0.001	<0.001
dC ~ Study_Area * Sex * Altered * Urban	4	54.06	<0.001	<0.001

AIC results for model selection used to identify the best model(s) to predict the effect of land use and sex on anthropogenic food consumption in individual coyotes. “dC” = average δ^13^C value of a coyote whisker; “Study_Area” = urban, suburban, or rural study area; “Altered” = percent of buffer zone that is altered open space (e.g., golf courses, cemeteries, city parks); “Sex” = male or female; “Urban” = percent of buffer zone that is urban land (commercial/industrial and residential land uses); “Humans” = human density (1,000 people/km^2^) in buffer zone. S3 Table contains a detailed distribution of land use variables for each buffer zone.

**Table 4:** AIC results for predicting nitrogen enrichment.

Model	K	ΔAIC	ωAIC	Likelihood
dN ~ Humans	1	0	0.929	1.000
dN ~ Study_Area * Sex + Humans	3	5.88	0.049	0.053
dN ~ Sex	1	9.24	0.009	0.010
dN ~ Study_Area	1	10.02	0.006	0.007
dN ~ Study_Area * Sex * Humans	3	10.35	0.005	0.006
dN ~ Study_Area * Sex	2	14.98	0.001	0.001
dN ~ Study_Area * Sex + Urban	3	17.27	< 0.001	< 0.001
dN ~ Study_Area * Sex + Altered	3	17.48	< 0.001	< 0.001
dN ~ Study_Area * Sex * Altered	3	20.01	< 0.001	< 0.001
dN ~ Study_Area * Sex * Urban	3	20.52	< 0.001	<0.001
dN ~ Study_Area * Sex * Altered * Humans	4	38.20	< 0.001	<0.001
dN ~ Study_Area * Sex * Altered * Urban	4	53.51	< 0.001	<0.001

AIC results for model selection used to identify the best model(s) to predict the effect of land use and sex on anthropogenic food consumption in individual coyotes. “dC” = average δ^13^C value of a coyote whisker; “Study_Area” = urban, suburban, or rural study area; “Altered” = percent of buffer zone that is altered open space (e.g., golf courses, cemeteries, city parks); “Sex” = male or female; “Urban” = percent of buffer zone that is urban land (commercial/industrial and residential land uses); “Humans” = human density (1,000 people/km^2^) in buffer zone. S3 Table contains a detailed distribution of land use variables for each buffer zone.

**Fig 5:**
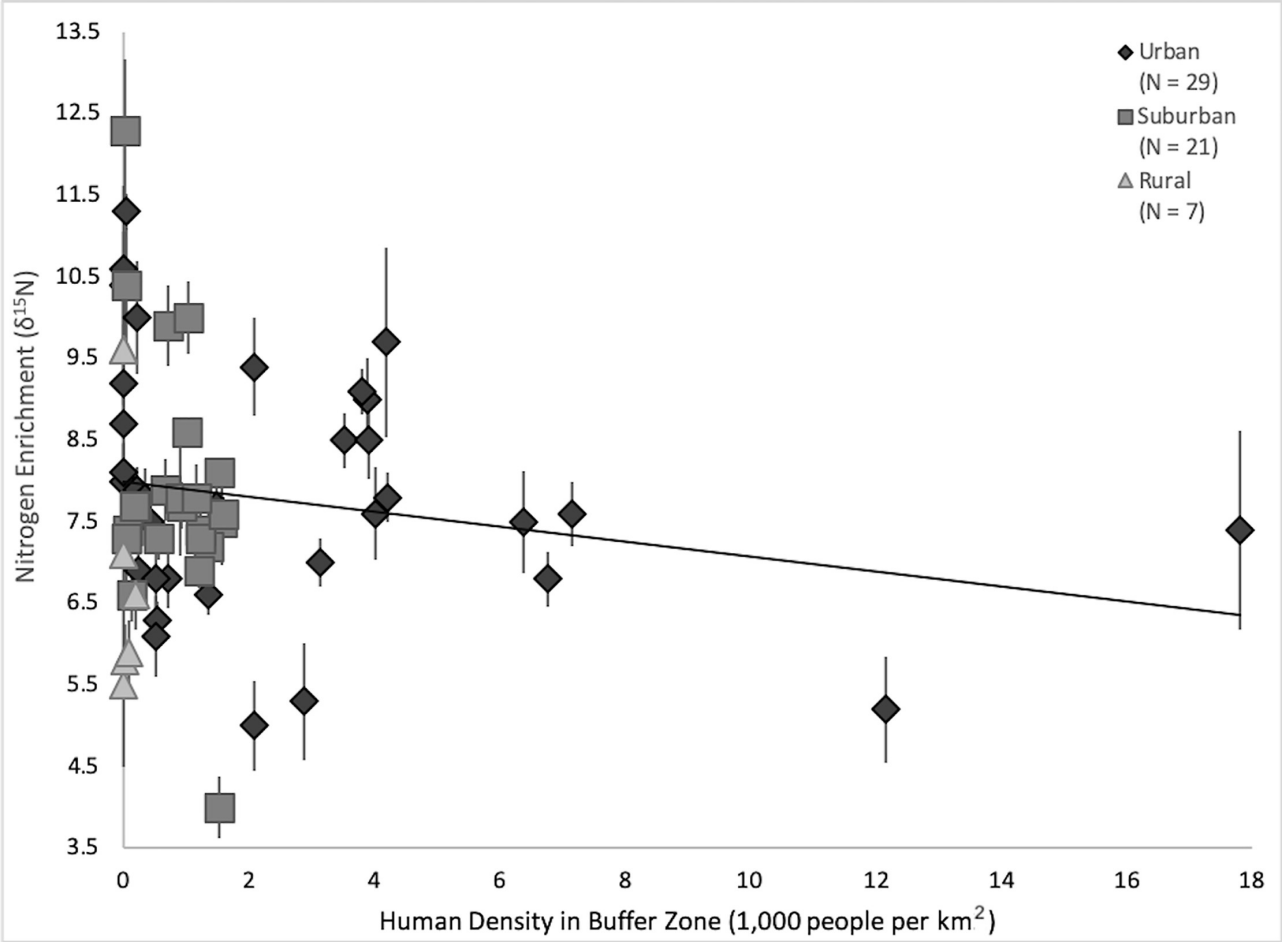
Human density negatively affects nitrogen enrichment of coyote vibrissae. The density of humans in the buffer zone around a coyote vibrissae collection site and the δ^15^N signature of the vibrissae. *Solid black line* represents a linear best-fit model. Sample sizes in the legend refer to the number of coyote vibrissae sampled from each study area.

Study area was a significant predictor of carbon isotope enrichment (*F*_2,58_ = 24.67, *p* < 0.001): rural individuals were the least enriched (δ^13^C = –21.6‰ ± 1.0‰ SD), followed by suburban individuals (–20.2‰ ± 1.2‰), and urban individuals were most enriched (–18.0‰ ± 1.7‰). Human density was a significant negative predictor of nitrogen isotope enrichment (*F*_1,59_ = 9.67, *p* = 0.003; Fig 5).

Variance component analysis of coyote vibrissa subsamples showed that variation in δ^13^C and δ^15^N values (i.e., niche width) increased with increasing urbanization (Table 5). Variation within individuals accounted for 84% of the variation in δ^13^C in urban coyotes, 61% of the variation in δ^13^C in suburban coyotes, and 52% of the variation in in δ^13^C rural coyotes. Variation in δ^15^N within individuals was similar across the levels of urbanization: the within-individual component explained 95% of variation in urban coyotes, 93% of variation in suburban coyotes, and 93% of variation in rural coyotes.

**Table 5:** Variance components of coyote whisker samples.

	Within-Individual Variance		Total Variance	
	δ^13^C	δ^15^N	δ^13^C	δ^15^N
Rural	24.8	374.1	47.8	400.6
Suburban	531.2	1,171.4	874.4	1,256.5
Urban	1,342.6	2,665.9	1,596.6	2,801.9

Calculated variances (*s*^*2*^) within individual coyote subsamples and the total variance of all subsamples.

## Discussion

Consumption of anthropogenic food resources was determined by which study area a coyote was found in, the season, and the amount of altered open space in the surrounding area. Results of the stable isotope analysis of vibrissae along with scat content data indicate that anthropogenic resource consumption increased as urbanization increased (Fig 1, Fig 4). The number of anthropogenic items consumed, such as domestic cats and human food, was dependent on the study area a scat transect or coyote was located in, but not related specifically to urban land cover, human density, or road density (Table 2). Nitrogen enrichment, generally regarded as representing the amount of protein in a consumer’s diet, decreased with increasing human density in the surrounding area (Table 4, Fig 5). Altered open space also had an effect on diet: scats from lines with more altered open space in their buffer zone had a reduced frequency of occurrence of anthropogenic items (Fig 2). The occurrence of anthropogenic items in scats was also affected by seasonality in suburban areas but not in urban areas (Fig 1). Suburban coyotes tended to increase their consumption of anthropogenic items, especially figs, during the dry season. Urban coyotes had relatively consistent diets with little change in the proportion of specific items throughout the year. This may be why individual specialization, as measured by variation in stable isotope signatures, was lower in the urban study area (Table 5).

It is somewhat unexpected that anthropogenic food item frequencies of occurrence in scat were not directly related to human density, road density, or urban land use, but rather the broader category of “study area”. Dietary protein assimilation (represented through nitrogen enrichment) was negatively related to human density. Though not statistically significant, coyotes with relatively low nitrogen enrichment also tended to have carbon enrichment values above the –20.3‰ threshold, indicating anthropogenic resource use. These coyotes may be hunting native prey less frequently, opting to use anthropogenic food because it could be found more easily in heavily-developed areas and is often reliable in space and time [68]. Our findings are consistent with those from other urban coyote studies that found increased consumption of anthropogenic resources with increasing proximity to urban areas [21,24,28,67,69–71]. The consumption of cats by urban coyotes was high (19.8% of scats), which is similar to another southern California study [69] but higher than typically reported by other urban coyote diet studies [21,24,28,70,71]. Cats receive supplemental food from their owners or feral colony caretakers, so their densities are more influenced by human housing density than prey density [72]. Feral and free-ranging cats may represent an abundant prey source for coyotes in urban environments. It is not surprising that the frequency of occurrence in scat and isotopically estimated biomass consumed of native mammals is lower in urban coyotes. Native rodent communities are negatively affected by urbanization but tend to be more common in areas with more shrub cover and habitat heterogeneity [22,23]. Urban coyotes have reduced access to patches with appropriate shrub cover for native rodent communities and have therefore switched to consuming human food and domestic cats. Lack of shrub cover may also be why access to altered open space lead to reduced frequency of occurrence of anthropogenic items in scats. Some categories of altered open space, such as golf courses, can increase local biodiversity when they are located near agricultural, residential, or urban areas [73]. Coyotes with access to altered open space may, therefore, have access to a more diverse prey base and consume anthropogenic foods less frequently.

The seasonal variation that we measured in suburban coyote scats was not from prey switching, but rather from seasonal expansion and contraction of niche breadth. Niche breadth expanded in the dry season since coyotes continued to consume native prey items while incorporating seasonally-available anthropogenic items. During the dry season, ornamental fruit and seeds were found more frequently (32% occurrence) compared to the wet season (16% occurrence). Ornamental plants, such as figs and grapes, tend to bear fruit in the dry season [74]. While this is also true of native plants, they were not commonly found in coyote scats and we were unable to detect seasonal variation in native plant consumption. Ornamental trees and shrubs bearing edible fruit may be more common in low-density suburban residential areas since tree and shrub cover tends to be inversely related to human population density [75]. This may explain why urban coyotes, that generally occur in more densely populated areas, consumed a smaller, more consistent amount of ornamental plants throughout the year. Figs are also C_3_ plants, much like the native plants that make up the base of the food chain in southern California. This may also be why fig consumption appears to increase in the suburban and rural study area based on stable isotope analysis. However, the stable isotope mixing model does not assign figs (the most common ornamental plant consumed) as making up a significant proportion of any population’s diet even though fig seeds occur frequently in scats in both study sites. Coyotes may not be able to fully digest plant material–if the isotopes are not assimilated into the coyote’s tissues (i.e., not digested or absorbed), this food item would not be apparent in the mixing model. The increased frequency of occurrence of reptiles in coyote scat in the dry season is also most likely related to increased availability. The most common reptilian prey items were large snakes (e.g., gopher snakes *Pituophis catenifer*), which reduce their activity during the cooler months of the year [76]. Coyotes encounter large snakes less frequently during the wet season when temperatures are cooler.

The amount of diet variation explained by within-individual variation in stable isotope values was 17% greater in the suburban and 62% greater in the urban study area compared to the rural study area. Rural and suburban coyotes appear to have similar levels of individual specialization (i.e., similar values of within-individual variance as a proportion of total variance) while urban coyotes had decreased specialization. Although urban coyote diets are broad and they consume native prey items, stable isotope modeling indicates the majority of their assimilated biomass comes from a C_4_ plant food chain (i.e., corn-based; consisting of human food, domestic cats, and pet food). It is possible that urban areas do not actually provide ecological opportunity in the form of increased resource heterogeneity. Rather, because prey diversity tends to drop as urbanization increases, one resource may replace another in an urban coyote’s diet. This would result in a shift of their dietary niche rather than an increase. American alligator (*Aligator mississippiensis*) populations confined to discreet, homogeneous lakes with low fish diversity had lower individual specialization than populations that inhabited an estuarine environment with diffuse boundaries between habitat types [11]. The lower individual specialization in urban coyotes may be due to minimal natural habitat availability, a predominantly urbanized landscape, the lack of common natural foods, and the high availability of anthropogenic items and human commensals. The availability of anthropogenic items may also be the cause of the lack of seasonal variation in urban scat composition. The scat niche width of suburban coyotes was 43% smaller than urban coyotes in the wet season and 18% smaller in the dry season. Rural coyotes had the smallest isotopic niche width and smallest within-individual variance. However, this may be due to a small sample size of rural individuals (N = 7). The isotopic niche width of suburban coyotes was smaller than urban coyotes, but within-individual variance was also lower in suburban areas. This could indicate that suburban coyotes are specializing on resources. Newsome et al. (2015) found some evidence for individual specialization in coyotes in urban forest preserves in Chicago, which approximates a suburban environment. Therefore, it may be suburban areas, where coyotes have access to both natural prey items and anthropogenic items, which provide ecological opportunity and promote individual specialization as expected under the niche variation hypothesis.

The inclusion of both traditional scat methods and stable isotope analysis help to produce the most accurate picture possible of coyote diets short of direct observations. Both methods showed a trend toward increasing anthropogenic item consumption and concurrent decrease in natural prey item consumption with increasing urbanization. In addition, both methods demonstrated that “study area” was the best predictor for determining anthropogenic item consumption rather than specifically human density, road density, or land cover. The exception was nitrogen enrichment, which was negatively related to human density. Scat analysis can over-represent rare food items and the calculated niche breadth of a population [54]. In addition, coyote scats that consisted of only human-related food items may have been misidentified as domestic dog and not collected. Our reported frequencies of pet food and food-related trash are most likely underestimated. However, the incorporation of stable isotopes should help reduce these biases. Stable isotope signatures represent overall assimilated biomass, which reduces the perceived importance of rare food items. Stable isotopes can also reliably discern anthropogenic versus natural food items in this system. Individual coyotes consuming large amounts of C_4_ plant-based food have distinct carbon signatures from coyotes that consume C_3_ plant-based food (i.e., native plants and mammals). It is possible that anthropogenic subsidies are more easily digestible and therefore more likely to be incorporated into a coyote’s tissues than a native prey item. This could bias the stable isotope models to underrepresent actual consumption rates of native prey items. However, scat data can help to elucidate consumption rates of native prey items. This is especially true where several food items are isotopically undifferentiated but easily distinguishable in scat. Scat data are also important for determining seasonal differences. Vibrissae grow over multiple months and may span different seasons, which means they are an average of long-term diet. Scats, on the other hand, represent individual meals that can be averaged over much shorter time spans (e.g., weeks). The power of detection of isotope analysis paired with the precision of scat analysis provides a complete picture of diet that takes into account long-term individual differences and seasonal population variation.

Our research provides one of the first comprehensive diet studies of coyotes living in the urban core of a large North American city. We provide evidence that “urban” and “suburban” are not synonymous to coyotes. Future work on highly urban carnivores could provide clearer patterns of how carnivore ecology changes across the full gradient of urbanization. Knowledge of food resource use can inform management efforts and prevent human-wildlife conflict associated with anthropogenic subsidies. In this particular system, unsecure garbage and ornamental fruits are important diet items that could be removed to make an area less attractive to coyotes. Coyotes with access to altered open space may also be less prone to conflict over anthropogenic resources, which could help target management efforts to coyotes in areas without any altered open space. Our research also provides evidence for individual specialization for coyotes living in rural and suburban environments. Approximately half of the variation in their diets was explained by differences between individuals, indicating that individuals maintained distinct diets from each other for at least the time span represented by their vibrissa (likely weeks to months). Coyotes with access to a greater variety of resources showed higher levels of inter-individual specialization, as predicted under the niche variation hypothesis. Suburban coyotes also altered their diets based on the season. Coyotes that have a broad overall diet may be seasonal specialists. This could be further teased apart if the growth rate of coyote vibrissae was known, allowing estimation of the time period covered by each vibrissa segment sampled. Further work should also be done to quantify the availability of food sources in urban and suburban areas. Accurate estimates of food availability (the density of populations of prey animals plus approximations of the availability of anthropogenic subsidies) would help create a more complete picture of urban coyote food preferences. Dramatic differences in diet likely influence important wildlife community interactions and could lead to the evolution of local adaptation in populations of urban wildlife.

## Supporting information

S1 Table Occurrence of diet items in coyote scats. The occurrences of food items in 3,147 coyote scats, categorized by city and season and identified to taxonomic family where possible. Within each category, N = the total number of scats, n_*i*_ = the number of scats containing a food item, FO is frequency of occurrence, and PO is percent occurrence. A niche breadth statistic (*B*) is also presented for each category (see text). Within each food item category, identified items are listed in descending order of FO across all sites.
(DOCX)

S2 Table Characteristics of 69 coyote vibrissa collected in Los Angeles and Ventura counties. *N* refers to the number of subsamples for each vibrissa. Average and standard deviation of each vibrissa’s δ^13^C and δ^15^N signatures are given.
(DOCX)

S3 Table Land use in the coyote vibrissa buffer zones. Land use and human development characteristics in 29 urban, 21 suburban, and 7 rural coyote buffer zones. Spatial analysis was done in ArcMap Desktop 10.6 (Esri, Inc.; Redlands, CA) using [49].
(DOCX)
