# Supplementary material for: Effects of urbanization on resource use and individual specialization in coyotes (Canis latrans) in southern California
Source: PLoS One. 2020 Feb 5;15(2):e0228881. doi: 10.1371/journal.pone.0228881 (PMC7001990; doi:10.1371/journal.pone.0228881)
Supplement: S1 Table — The occurrences of food items in 3,147 coyote scats, categorized by city and season and identified to taxonomic family where possible. Within each category, N = the total number of scats, ni = the number of scats containing a food item, FO is frequency of occurrence, and PO is percent occurrence. A niche breadth statistic (B) is also presented for each category (see text). Within each food item category, identified items are listed in descending order of FO across all sites. (DOCX) [file pone.0228881.s001.docx]

| **Study Area** | **Urban**  N = 1,541 | | | | | | **Suburban**  N = 1,606 | | | | | | | |  |
| --- | --- | --- | --- | --- | --- | --- | --- | --- | --- | --- | --- | --- | --- | --- | --- |
| **Season** | **Wet**  N = 693 | | | **Dry**  N = 848 | | | **Wet**  N = 788 | | | **Dry**  N = 818 | | | | |  |
|  | n*_i_* | FO | PO | n*_i_* | FO | PO | n*_i_* | FO | PO | n*_i_* | | FO | PO | |  |
| **Mammals** | **327** | **47.2%** | **30.4%** | **429** | **50.6%** | **29.7%** | **688** | **87.3%** | **64.7%** | **664** | | **81.2%** | **51.5%** | |  |
| Rabbit | 135 | 19.5% | 10.4% | 143 | 16.9% | 9.0% | 432 | 54.8% | 32.1% | 339 | | 42.7% | 21.1% | |  |
| Rodent^a^ | 152 | 21.9% | 12.8% | 177 | 20.9% | 12.2% | 317 | 40.2% | 27.3% | 367 | | 44.9% | 25.8% | |  |
| *Pocket Gopher* | 58 | 8.4% | 4.4% | 75 | 8.8% | 4.3% | 115 | 14.6% | 8.5% | 106 | | 13.0% | 6.4% | |  |
| *Squirrel* | 47 | 6.8% | 3.6% | 68 | 8.0% | 4.3% | 82 | 10.4% | 6.1% | 178 | | 21.8% | 10.7% | |  |
| *Native Mouse^b^* | 20 | 2.9% | 1.5% | 19 | 2.2% | 1.2% | 45 | 5.7% | 3.3% | 43 | | 5.3% | 2.6% | |  |
| *Woodrat* | 13 | 1.9% | 1.0% | 10 | 1.2% | 0.6% | 67 | 8.5% | 5.0% | 56 | | 6.9% | 3.4% | |  |
| *Pocket Mouse* | 1 | 0.1% | 0.1% | 0 | - | - | 15 | 1.9% | 1.1% | 9 | | 1.1% | 0.5% | |  |
| *Vole* | 0 | - | - | 0 | - | - | 12 | 1.5% | 0.9% | 16 | | 2.0% | 1.0% | |  |
| *Unidentified Rodent* | 28 | 4.0% | 2.1% | 22 | 2.6% | 1.4% | 31 | 3.9% | 2.3% | 20 | | 2.4% | 1.2% | |  |
| Carnivores^c^ | 34 | 4.9% | 2.6% | 58 | 6.8% | 3.5% | 11 | 1.4% | 0.9% | 10 | | 1.2% | 0.6% | |  |
| *Raccoon* | 11 | 1.6% | 0.8% | 14 | 1.7% | 0.9% | 6 | 0.8% | 0.4% | 2 | | 0.2% | 0.1% | |  |
| *Skunk* | 9 | 1.3% | 0.7% | 13 | 1.5% | 0.8% | 3 | 0.4% | 0.3% | 3 | | 0.4% | 0.2% | |  |
| *Unidentified Carnivore* | 14 | 2.0% | 1.1% | 28 | 3.3% | 1.8% | 3 | 0.4% | 0.2% | 5 | | 0.6% | 0.3% | |  |
| Opossum | 6 | 0.9% | 0.5% | 2 | 0.2% | 0.1% | 0 | - | - | 5 | | 0.6% | 0.3% | |  |
| Deer | 0 | - | - | 0 | - | - | 7 | 0.9% | 0.5% | 7 | | 0.9% | 0.4% | |  |
| Shrews | 0 | - | - | 0 | - | - | 1 | 0.1% | 0.1% | 1 | | 0.1% | 0.1% | |  |
| Unidentified Mammals | 54 | 7.8% | 4.1% | 78 | 9.2% | 4.9% | 52 | 6.6% | 3.9% | 53 | | 6.5% | 3.2% | |  |
| **Anthropogenic** | **458** | **66.1%** | **43.9%** | **545** | **64.3%** | **42.9%** | **220** | **27.9%** | **18.3%** | **374** | | **45.7%** | **25.8%** | |  |
| *Ornamental Fruit* | 170 | 24.5% | 13.0% | 232 | 27.4% | 14.6% | 126 | 16.0% | 9.4% | 260 | | 31.8% | 15.7% | |  |
| *Trash* | 155 | 22.4% | 11.9% | 180 | 21.2% | 11.3% | 40 | 5.0% | 3.0% | 56 | | 6.9% | 3.4% | |  |
| *Domestic Cat* | 150 | 21.6% | 11.5% | 156 | 18.4% | 9.8% | 27 | 3.4% | 2.0% | 34 | | 4.2% | 2.1% | |  |
| *Pet Food* | 29 | 4.2% | 2.2% | 21 | 2.5% | 1.3% | 9 | 1.1% | 0.7% | 20 | | 2.4% | 1.2% | |  |
| *Rat* | 28 | 4.0% | 2.1% | 40 | 4.7% | 2.5% | 23 | 2.9% | 1.7% | 15 | | 1.8% | 0.9% | |  |
| *House Mouse* | 14 | 2.0% | 1.1% | 7 | 0.8% | 0.4% | 3 | 0.4% | 0.2% | 6 | | 0.7% | 0.4% | |  |
| *Domestic Dog* | 10 | 1.4% | 0.8% | 12 | 1.4% | 0.8% | 3 | 0.4% | 0.2% | 1 | | 0.1% | 0.1% | |  |
| *Eastern Fox Squirrel* | 6 | 0.9% | 0.5% | 17 | 2.0% | 1.1% | 13 | 1.7% | 1.0% | 26 | 3.2% | | | 1.6% | |
| *Domestic Rabbit* | 4 | 0.5% | 0.2% | 3 | 0.3% | 0.2% | 0 | - | - | 0 | - | | | - | |
| *Domestic Chicken* | 2 | 0.3% | 0.2% | 2 | 0.2% | 0.1% | 1 | 0.1% | 0.1% | 5 | 0.6% | | | 0.3% | |
| *Unidentified Domestic Animal* | 8 | 1.2% | 0.6% | 15 | 1.8 | 0.9% | 1 | 0.1% | 0.1% | 5 | 0.6% | | | 0.3% | |
| **Invertebrates** | **110** | **15.9%** | **8.4%** | **178** | **21.0%** | **11.2%** | **101** | **12.8%** | **7.5%** | **133** | | **16.3%** | **8.0%** | |  |
| **Birds** | **110** | **15.9%** | **8.4%** | **113** | **13.3%** | **7.1%** | **36** | **4.6%** | **2.7%** | **53** | | **6.5%** | **3.2%** | |  |
| **Reptiles** | **10** | **1.4%** | **0.8%** | **22** | **2.6%** | **1.4%** | **19** | **2.4%** | **1.4%** | **90** | | **11.0%** | **5.4%** | |  |
| **Native Fruit** | **7** | **1.0%** | **0.5%** | **15** | **1.8%** | **0.9%** | **7** | **0.9%** | **0.5%** | **15** | | **1.8%** | **0.9%** | |  |
| **Niche Breadth** | 7.23 | | | 7.31 | | | 4.04 | | | 5.98 | | | | |  |

^a.^“Rodent” does not include house mice (*Mus musculus*), rats (*Rattus* spp.), or eastern fox squirrels (*Sciurus niger*).

^b.^ “Native Mouse” includes deermice (*Peromyscus* spp.) and western harvest mice (*Reithrodontomys megalotis*).

^c.^ “Carnivores” does not include domestic cats (*Felis catus*) or domestic dogs (*Canis lupus familiaris*).
