# Supplementary material for: Effects of urbanization on resource use and individual specialization in coyotes (Canis latrans) in southern California
Source: PLoS One. 2020 Feb 5;15(2):e0228881. doi: 10.1371/journal.pone.0228881 (PMC7001990; doi:10.1371/journal.pone.0228881)
Supplement: S2 Table — N refers to the number of subsamples for each vibrissa. Average and standard deviation of each vibrissa’s δ13C and δ15N signatures are given. (DOCX) [file pone.0228881.s002.docx]

| **Coyote^a^** | ***N*** | **Length (mm)** | **dN** | **sd_N** | **dC** | **sd_C** |
| --- | --- | --- | --- | --- | --- | --- |
| CM09 | 8 | 49 | 9.4 | 0.6 | -18.9 | 0.6 |
| CM11 | 36 | 95 | 7.6 | 0.4 | -18.5 | 0.6 |
| CM12 | 20 | 73 | 7.5 | 0.4 | -18.0 | 0.6 |
| CM15 | 38 | 102 | 7.8 | 0.3 | -17.1 | 0.6 |
| CM16 | 16 | 59 | 7.5 | 0.6 | -15.4 | 0.7 |
| CM20 | 17 | 76 | 9.2 | 0.4 | -18.2 | 1.0 |
| CM21 | 14 | 68 | 8.0 | 0.5 | -16.4 | 0.5 |
| CM22 | 27 | 91 | 7.0 | 0.3 | -15.9 | 0.4 |
| CM23 | 18 | 68 | 9.7 | 1.2 | -18.5 | 0.7 |
| CM26 | 27 | 93 | 6.8 | 0.3 | -20.2 | 0.5 |
| CM30 | 21 | 70 | 8.7 | 0.4 | -16.2 | 0.7 |
| CM40 | 6 | 57 | 11.3 | 0.2 | -22.5 | 0.2 |
| CM42 | 14 | 60 | 7.6 | 0.6 | -17.5 | 0.4 |
| CM44 | 11 | 73 | 9.0 | 0.5 | -17.3 | 0.5 |
| CM45 | 14 | 65 | 7.7 | 0.3 | -16.1 | 0.3 |
| CM54 | 18 | 82 | 8.1 | 0.3 | -16.2 | 0.5 |
| CM61 | 23 | 82 | 10.6 | 1.0 | -18.9 | 0.7 |
| CM63 | 23 | 82 | 10.4 | 0.7 | -19.6 | 0.8 |
| CM98 | 15 |  | 6.6 | 0.2 | -22.1 | 0.4 |
| C144 | 4 | 39 | 5.2 | 0.7 | -18.0 | 0.6 |
| C145 | 16 | 74 | 5.3 | 0.7 | -17.9 | 0.6 |
| C146.c | 4 | 39 | 9.1 | 0.3 | -16.2 | 0.2 |
| C146.m | 23 | 82 | 7.4 | 1.2 | -18.5 | 0.7 |
| C148.c | 13 | 63 | 5.0 | 0.6 | -19.0 | 0.6 |
| C148.m | 17 | 77 | 8.5 | 0.3 | -17.3 | 0.7 |
| C149 | 19 | 83 | 8.5 | 0.5 | -15.9 | 0.9 |
| C150 | 4 | 42 | 10.0 | 0.7 | -20.4 | 0.3 |
| C151.c | 17 | 69 | 6.3 | 0.2 | -16.1 | 0.6 |
| C151.m | 21 | 63 | 6.1 | 0.5 | -16.1 | 1.1 |
| C153 | 15 | 82 | 7.9 | 0.3 | -20.6 | 0.8 |
| C154 | 14 | 64 | 6.9 | 0.3 | -20.0 | 0.7 |
| C155 | 15 | 63 | 6.8 | 0.4 | -19.4 | 0.6 |
| C156 | 18 | 71 | 7.8 | 0.4 | -19.1 | 1.0 |
| C157 | 9 | 58 | 6.8 | 0.7 | -17.0 | 1.6 |
| CM25 | 20 | 86 | 9.6 | 0.7 | -20.5 | 0.4 |
| CM62 | 17 | 70 | 9.6 | 0.3 | -20.8 | 0.7 |
| C131 | 5 | 45 | 6.6 | 0.4 | -23.1 | 0.2 |
| C135 | 3 | 28 | 5.5 | 1.0 | -20.6 | 0.6 |
| C140 | 5 | 46 | 5.8 | 0.4 | -21.4 | 0.8 |
| C142 | 16 | 58 | 7.1 | 0.4 | -22.4 | 0.4 |
| C143 | 14 | 65 | 5.9 | 0.4 | -22.4 | 0.8 |
| CM10 | 15 | 86 | 7.9 | 0.4 | -20.3 | 0.8 |
| CM33 | 20 | 80 | 9.9 | 0.5 | -20.8 | 0.4 |
| CM56 | 10 | 74 | 7.5 | 0.5 | -20.4 | 1.1 |
| CM60 | 22 | 81 | 7.4 | 0.4 | -18.6 | 0.8 |
| CM64 | 17 | 73 | 7.2 | 0.3 | -18.6 | 0.8 |
| CM66 | 26 | 99 | 7.7 | 0.3 | -20.6 | 0.9 |
| CM67 | 17 | 77 | 7.3 | 0.6 | -19.3 | 1.6 |
| CM70 | 20 | 80 | 7.3 | 0.3 | -20.8 | 0.5 |
| CM75 | 9 | 67 | 7.4 | 0.4 | -20.5 | 0.7 |
| CM76 | 14 | 50 | 10.0 | 0.4 | -20.8 | 0.5 |
| CM77 | 24 | 78 | 7.3 | 0.4 | -20.2 | 0.9 |
| CM78 | 16 | 71 | 7.7 | 0.4 | -19.6 | 0.5 |
| CM81 | 12 | 56 | 12.3 | 0.9 | -23.0 | 0.3 |
| CM83 | 11 | 80 | 8.1 | 0.2 | -20.0 | 0.7 |
| CM84 | 6 | 63 | 6.9 | 0.2 | -20.7 | 0.8 |
| CM87 | 9 | 56 | 8.6 | 0.2 | -21.6 | 0.6 |
| CM88 | 5 | 51 | 7.6 | 0.2 | -18.9 | 0.2 |
| CM89 | 11 | 63 | 7.7 | 0.3 | -20.9 | 0.8 |
| CM97 | 15 | 73 | 6.6 | 0.3 | -17.6 | 0.5 |
| CM101 | 13 |  | 10.4 | 0.7 | -22.9 | 0.4 |
| CM102 | 20 |  | 7.8 | 0.7 | -21.8 | 0.8 |
| C147 | 20 | 85 | 4.0 | 0.4 | -21.2 | 0.5 |
| C152.m | 19 | 96 | 7.8 | 0.4 | -18.8 | 0.9 |

^a.^ “CM” stands for “coyote mortality” and designates a coyote found dead (e.g., roadkill). “C” designates a coyote captured alive as part of a separate collaring and tracking study. If the same individual was sampled twice (e.g., C146), it will have a “.c” after its name to indicate the sample from the capture event, and a “.m” after its name to indicate the sample from the mortality event.
