# Supplementary material for: Effects of urbanization on resource use and individual specialization in coyotes (Canis latrans) in southern California
Source: PLoS One. 2020 Feb 5;15(2):e0228881. doi: 10.1371/journal.pone.0228881 (PMC7001990; doi:10.1371/journal.pone.0228881)
Supplement: S3 Table — Land use and human development characteristics in 29 urban, 21 suburban, and 7 rural coyote buffer zones. Spatial analysis was done in ArcMap Desktop 10.6 (Esri, Inc.; Redlands, CA) using [49]. (DOCX) [file pone.0228881.s003.docx]

| **Coyote^a^** | **Study_Area** | **Urban^b^** | **Altered^c^** | **Humans^d^** | **Sex^e^** |
| --- | --- | --- | --- | --- | --- |
| CM09 | Urban | 0.8153 | 0.1674 | 0.209 | F |
| CM11 | Urban | 0.9282 | 0.0052 | 0.716 | M |
| CM12 | Urban | 0.3025 | 0.1431 | 0.043 | M |
| CM15 | Urban | 0.7981 | 0.0513 | 0.422 | F |
| CM16 | Urban | 0.9979 | 0 | 0.638 | M |
| CM20 | Urban | 0.9086 | 0.0871 | 0 | F |
| CM21 | Urban | 0.9086 | 0.0871 | 0 | M |
| CM22 | Urban | 0.5662 | 0.0412 | 0.314 | M |
| CM23 | Urban | 0.9619 | 0.0381 | 0.418 | F |
| CM26 | Urban | 0.9900 | 0.0067 | 0.677 | M |
| CM30 | Urban | 0.9086 | 0.0871 | 0 | M |
| CM40 | Urban | 0.9885 | 0.0000 | 0.004 | M |
| CM42 | Urban | 0.8837 | 0.0264 | 0.401 | F |
| CM44 | Urban | 0.8824 | 0.0025 | 0.389 | M |
| CM45 | Urban | 0.9870 | 0.0055 | 0.148 | F |
| CM54 | Urban | 0.9086 | 0.0871 | 0 | F |
| CM61 | Urban | 0.9086 | 0.0871 | 0 | M |
| CM63 | Urban | 0.9086 | 0.0871 | 0 | M |
| C144 | Urban | 0.9266 | 0.0131 | 1.215 | F |
| C145 | Urban | 0.8116 | 0.0382 | 0.287 | M |
| C146.c | Urban | 0.8928 | 0.0029 | 0.381 | F |
| C146.m | Urban | 0.9584 | 0.0345 | 1.779 | F |
| C148.c | Urban | 0.8145 | 0.1681 | 0.209 | M |
| C148.m | Urban | 0.9108 | 0.0577 | 0.353 | M |
| C149 | Urban | 0.8689 | 0.0025 | 0.390 | F |
| C150 | Urban | 0.1952 | 0.0530 | 0.021 | F |
| C151.c | Urban | 0.1842 | 0.0089 | 0.054 | F |
| C151.m | Urban | 0.2989 | 0.0069 | 0.052 | F |
| C153 | Urban | 0.0902 | 0.1724 | 0.022 | M |
| C154 | Urban | 0.1019 | 0.1764 | 0.023 | F |
| C155 | Urban | 0.5156 | 0.0000 | 0.072 | F |
| C156 | Urban | 0.2276 | 0.0088 | 0.034 | M |
| C157 | Urban | 0.1783 | 0.0048 | 0.052 | M |
| CM25 | Rural | 0 | 0 | 0 | F |
| CM62 | Rural | 0.0301 | 0 | 0.010 | F |
| C131 | Rural | 0.2380 | 0.5121 | 0.190 | F |
| C135 | Rural | 0.0442 | 0.1897 | 0.010 | F |
| C140 | Rural | 0.0401 | 0.4652 | 0.020 | M |
| C142 | Rural | 0.0225 | 0.7392 | 0.010 | M |
| C143 | Rural | 0.2669 | 0.3018 | 0.090 | M |
| CM10 | Suburban | 0.4690 | 0.1368 | 0.066 | M |
| CM33 | Suburban | 0.5128 | 0.1422 | 0.072 | M |
| CM56 | Suburban | 0.6774 | 0.0133 | 0.157 | F |
| CM60 | Suburban | 0.1310 | 0.0522 | 0.007 | F |
| CM64 | Suburban | 0.7010 | 0.0116 | 0.135 | F |
| CM66 | Suburban | 0.2372 | 0.0106 | 0.019 | M |
| CM67 | Suburban | 0.1100 | 0.0120 | 0.005 | M |
| CM70 | Suburban | 0.2892 | 0.0323 | 0.056 | F |
| CM75 | Suburban | 0.5597 | 0.0179 | 0.123 | F |
| CM76 | Suburban | 0.4398 | 0.0370 | 0.103 | M |
| CM77 | Suburban | 0.5597 | 0.0179 | 0.123 | M |
| CM78 | Suburban | 0.1990 | 0.0111 | 0.017 | F |
| CM81 | Suburban | 0.0530 | 0.1164 | 0.003 | F |
| CM83 | Suburban | 0.6328 | 0.0253 | 0.153 | M |
| CM84 | Suburban | 0.5602 | 0.2011 | 0.121 | F |
| CM87 | Suburban | 0.5911 | 0.0083 | 0.101 | F |
| CM88 | Suburban | 0.7522 | 0.0645 | 0.158 | M |
| CM89 | Suburban | 0.2566 | 0.0140 | 0.092 | M |
| CM97 | Suburban | 0.1482 | 0.0095 | 0.014 | M |
| C147 | Suburban | 0.6043 | 0.0133 | 0.152 | F |
| C152.m | Suburban | 0.6128 | 0.0042 | 0.116 | F |

^a.^ “CM” stands for “coyote mortality” and designates a coyote found dead (e.g., roadkill). “C” designates a coyote captured alive as part of a separate collaring and tracking study. If the same individual was sampled twice (e.g., C146), it will have a “.c” after its name to indicate the sample from the capture event, and a “.m” after its name to indicate the sample from the mortality event.

^b.^ Proportion of the buffer zone’s surface area that was classified as commercial/industrial or residential land use [49].

^c.^ Proportion of the buffer zone’s surface area that was classified as agriculture or developed parks/other open space land use [49].

^d.^ Human density in the buffer zone in 10,000 people per km^2^.

^e.^ “F” designates a female, “M” designates a male.
